# Caffeine supplementation induces higher IL-6 and IL-10 plasma levels in response to a treadmill exercise test

**DOI:** 10.1186/s12970-020-00375-4

**Published:** 2020-09-09

**Authors:** Lluis Rodas, Sonia Martinez, Antoni Aguilo, Pedro Tauler

**Affiliations:** 1 Research Group on Evidence, Lifestyles & Health, Department of Fundamental Biology and Health Sciences, Research Institute on Health Sciences (IUNICS). University of the Balearic Islands, Crta de Valldemossa, Km 7.5, E-07122 Palma, Spain; 2 Research Group on Evidence, Lifestyles & Health, Department of Nursing and Physiotherapy, Research Institute on Health Sciences (IUNICS). University of the Balearic Islands, Crta de Valldemossa, Km 7.5, E-07122 Palma, Spain; 3 Health Research Institute of the Balearic Islands (IdISBa), Palma, Spain

**Keywords:** Exercise, Caffeine, Inflammation, Cytokines, Adrenaline

## Abstract

**Background:**

An acute bout of exercise induces an inflammatory response characterized by increases in several cytokines. Caffeine ingestion could modify this inflammatory response. The aim of this study was to determine the effects of caffeine supplementation on plasma levels of cytokines, mainly IL-10 and IL-6, in response to exercise.

**Methods:**

In a randomized, crossover, double-blinded study design, thirteen healthy, well-trained recreational male athletes performed, on two different occasions, a treadmill exercise test (60 min at 70% VO_2_max) after ingesting 6 mg/kg body mass of caffeine or placebo. Blood samples were taken before exercising, immediately after finishing and 2 h after finishing the exercise. Plasma concentrations of IL-10, IL-6, IL-1β, IL-1ra, IL-4, IL-8, IL-12 and IFN-γ, adrenaline, cortisol and cyclic adenosine monophosphate (cAMP) were determined. The capacity of whole blood cultures to produce cytokines in response to endotoxin (LPS) was also determined. Changes in blood variables were analyzed using a time (pre-exercise, post-exercise, recovery) x condition (caffeine, placebo) within-between subjects ANOVA with repeated measures.

**Results:**

Caffeine supplementation induced higher adrenaline levels in the supplemented participants after exercise (257.3 ± 53.2 vs. 134.0 ± 25.7 pg·mL^− 1^, *p* = 0.03) and higher cortisol levels after recovery (46.4 ± 8.5 vs. 32.3 ± 5.6 pg·mL^− 1^, *p* = 0.007), but it did not influence plasma cAMP levels (*p* = 0.327). The exercise test induced significant increases in IL-10, IL-6, IL-1ra, IL-4, IL-8, IL-12 and IFN-γ plasma levels, with IL-6 and IL-10 levels remaining high after recovery. Caffeine supplementation influenced only IL-6 (3.04 ± 0.40 vs. 3.89 ± 0.62 pg·mL^− 1^, *p* = 0.003) and IL-10 (2.42 ± 0.54 vs. 3.47 ± 0.72 pg·mL^− 1^, *p* = 0.01) levels, with higher concentrations after exercise in the supplemented condition. No effect of caffeine was observed on the in vitro stimulated cytokine production.

**Conclusions:**

The results of the present study indicate a significant influence of caffeine supplementation increasing the response to exercise of two essential cytokines such as IL-6 and IL-10. However, caffeine did not influence changes in the plasma levels of other cytokines measured and the in vitro-stimulated cytokine production.

## Background

Caffeine is a member of the methylxanthine family of drugs. Several studies have reported improvements in exercise performance after the ingestion of 3–6 mg/kg body mass of caffeine, particularly in endurance events when metabolic mechanisms are not limiting [1, 2]. The ergogenic and stimulatory effects of caffeine, its presence in common products such as coffee, tea, and sport nutrition products, and its removal from the doping list in 2008 have contributed to its widespread consumption among athletes [3, 4].

The inflammatory response to an acute bout of exercise is characterized by increases in pro- and anti-inflammatory cytokines [5]. Within this response, interleukin 10 (IL-10) has been suggested to play an essential role by limiting the production of pro-inflammatory factors [6, 7]. Furthermore, it has been reported that IL-10 enhances myogenesis [8], giving more importance to the role of IL-10 in the response to exercise. However, the inflammatory response to exercise includes increases of other cytokines, mainly IL-6 [5, 9]. In this regard, it has been reported that high IL-6 concentrations in response to exercise prevent increases in pro-inflammatory cytokines such as TNF-α and induce the production of anti-inflammatory cytokines such as IL-10 and IL-1ra [10], conferring anti-inflammatory properties to that response [9, 11].

At physiological doses, caffeine has been reported to mainly exert its effects via adenosine receptor antagonism, and in this manner acts as a powerful central nervous system stimulator and upregulates the synthesis of catecholamines such as adrenaline and noradrenaline [2, 4]. Adenosine receptors are associated with intracellular pathways that influence the production of cyclic adenosine monophosphate (cAMP) [12], a second messenger with immunomodulatory properties [13] that induces, among other cytokines, IL-10 production [14]. Therefore, any change in cAMP concentration induced by caffeine could modify this inflammatory status, mainly by modifying IL-10 production. It has also been reported that adrenaline can influence IL-6 levels by reducing its clearance by the liver [15].

It is worth mentioning that in previous studies, we reported that caffeine supplementation increases plasma IL-10 and IL-6 concentrations in response to exercise [16, 17]. However, these studies were performed in two 15-km run competitions, with a lack of control on some important conditions such as participant exercise intensity and environmental conditions. The aim of this study was to determine the effects of caffeine supplementation on plasma levels of IL-10 and IL-6 in response to a nonstrenuous exercise test performed on a treadmill. To complete the description of the inflammatory response, changes induced on additional plasma cytokines such as IL-12, IL-1ra and IL-1β were also measured. The capacity of whole blood cultures to produce cytokines in response to endotoxin (LPS) was also determined. Considering previous results, the main hypothesis of the study was that caffeine could increase IL-10 and IL-6 circulatory concentrations in response to exercise.

## Materials and methods

### Participants

Thirteen healthy, well-trained, recreational male athletes took part in the study. All the subjects were informed of the purpose and demands of the study before giving their written consent to participate. The protocol was in accordance with the Declaration of Helsinki for research of human subjects and was approved by the Balearic Islands Clinical Investigation Ethics Committee (reference IB 2399/14 PI).

Participants were enrolled after fulfilling all inclusion criteria and possessing none of the exclusion criteria. Subjects could be included if they were currently healthy, consumed caffeine regularly in any form (coffee, tea, soda, supplements, etc.), were between 20 and 45 years old, and engaged in at least 8 h of training or competition per week. Exclusion criteria were: smokers, use of analgesic or anti-inflammatory drugs within the previous 2 weeks, habitual alcohol consumers, and presence of injury or illness incompatible with the sport practice or caffeine intake. Fourteen subjects were initially recruited, but one of them was dropped from the study, as he had only performed the main exercise in the placebo condition due to lack of availability. Table 1 shows the main characteristics of participants in the study. Participants in the study did not consume caffeine supplements, and they reported a habitual caffeine intake similar to the one that would result from 1 to 2 cups of coffee.

**Table 1:** General characteristics of participants

Parameter	
Age (yr)	36.1 ± 6.4
Weight (kg)	75.6 ± 7.4
Height (cm)	177.4 ± 6.0
BMI (kg·m^− 2^)	24.1 ± 2.2
% fat (Faulkner)	12.6 ± 2.3
Resting heart rate (beats·min^−1^)	57.0 ± 4.8
Physical activity (MET-h·wk.^− 1^)	111.1 ± 48.9
Physical activity (sessions·wk.^− 1^)	13.5 ± 3.1
Habitual caffeine intake (mg·day^− 1^)	142.6 ± 96.5
Energy intake (kcal)	2917.1 ± 357.8
CHO (% total energy)	42.0 ± 3.3
Proteins (% total energy)	17.0 ± 2.3
Lipids (% total energy)	41.0 ± 4.2
VO_2_max (ml·kg^−1^·min^− 1^)	54.2 ± 6.1
Speed 70%VO_2_max (km·h^− 1^)	12.3 ± 1.3

The values are the mean ± S.D. (*n* = 13)

*BMI* body mass index, *MET* metabolic equivalents, *wk.* week

### Experimental design

A randomized, crossover, double-blinded study of supplementation with caffeine was performed. At 8–10 days before the beginning of the study, each subject performed a continuous incremental exercise test on a treadmill (H-P Cosmos, Pulsar 3, Nussdorf-Traunstein, Germany) until volitional exhaustion to determine their maximal oxygen uptake (VO_2_max). The participants warmed up for 3 min at 4 km/h before starting the test. The test began at 6 km/h, and the participant work rate was increased by 1 km/h every minute until exhaustion. Each test was ended when added work did not increase or decrease the oxygen consumption, and the resulting value was recorded as the VO_2_max. Expired gas was continuously analyzed (H-P Cosmos, Jaeger-MasterScreen CPX, Nussdorf-Traunstein, Germany), and heart rates (HR) were measured continuously using short-range radio telemetry (Polar Beat; Polar Electro, Oy, Finland). Level running speeds equivalent to 70% VO_2_max were subsequently calculated via regression equations from the VO_2_-running speed relationship. Before the exercise test, participants’ height, weight and skinfolds required to measure body fat mass using the Faulkner equation were measured (triceps, abdominal, subscapular, and suprailiac).

Questionnaires to determine habitual nutritional and caffeine intake, as well as the amount of physical activity performed, were filled out while the participant was in the laboratory (by the participant himself) or were given to be completed and turned in on the first main trial day. Habitual physical activity was determined by using the International Physical Activity Questionnaire (IPAQ) [18], thus providing quantitative information on training loads in metabolic equivalent (MET)-h/week. Habitual nutritional intake was assessed using a self-reported 7-day food record. Mean energy, carbohydrate, protein, and lipid contents for each participant’s diet were calculated using commercial software (Nutrisalud; CSG Software, Huesca, Spain) based on Spanish food composition tables. Furthermore, habitual caffeine intake was measured with a self-reported caffeine consumption questionnaire used by our group [16, 17].

Finally, each subject was given a comprehensive list of caffeine-containing foods and drinks and was instructed to abstain from these products during the 24 h preceding each exercise trial. The subjects were not allowed to use analgesic or anti-inflammatory drugs during the study protocol, and they were instructed to maintain their habitual diet, to refrain from alcohol intake and not to participate in any sporting activity during the 48 h preceding each main experimental trial.

For the main exercise trials, on two occasions 1 week apart, the subjects reported to the laboratory at the same hour and were randomly assigned to either the caffeine or placebo trial in a crossover random block design. The subjects were then required to empty their bladder before body mass (in shorts only) was recorded. After sitting quietly for 10 min, an initial resting blood sample was obtained from an antecubital vein by venipuncture. Following blood sampling, in the caffeine trial subjects were given 6 mg·kg^− 1^ body mass of caffeine dissolved in 200 ml of fruit juice drink; in the placebo trial, the subjects were given the same volume of only the fruit juice. Both drinks were matched to be similar in taste and appearance. A 24-h recall was performed to ensure that nutritional intake was similar before each trial (results not shown) and within the participants’ habitual nutritional intake. After resting quietly in the laboratory for 1 h and a short warm-up, subjects began running on the treadmill at the speed equivalent to 70% VO_2_max for 60 min. Heart rates were continuously recorded, and expired air was analyzed every 20 min thereafter for control of intensity. An additional venous blood sample was obtained at immediately post-exercise before body mass (in shorts only) was recorded again. A final venous blood sample was obtained at 2 h post-exercise. For all samples, 12 ml of blood was collected, and samples were obtained with the subject in a seated position. For both trials, subjects could drink water ad libitum, with the water intake during the test measured. No other fluid or food intake was allowed until the blood sample had been collected at 2 h post-exercise. Laboratory conditions were 22.0 ± 0.6 °C and 54.2 ± 6.1% relative humidity.

### Blood sampling and measurements

Seated venous blood samples were collected in suitable vacutainers with ethylenediaminetetraacetic acid (EDTA) or heparin as the anticoagulant. Hematocrit and hemoglobin were determined in the EDTA sample using a hematology analyzer (Horiba ABX Pentra 60, Diagnostics) for estimating plasma volume [19]. Within 30 min after blood collection, plasma was obtained by centrifugation (15 min, 1000×g, 4 °C) of the EDTA-blood samples. These plasma samples were stored at − 70 °C until measurements were performed. Concentrations of cytokines (IL-10, IL-6, IL-8, IL-1ra, IL-4, IL-1β, IL-12 and IFN-γ, caffeine, adrenaline, cortisol and cAMP were measured in the EDTA plasma samples.

Whole heparinized blood was incubated with 10 ng/ml lipopolysaccharide (LPS, *Escherichia coli* serotype 055:B5; Sigma, St Louis, MO, USA) dissolved in culture medium (RPMI-1640 Medium, Sigma, St Louis, MO, USA), or with the same volume of culture medium (spontaneous production) for 24 h at 37 °C. Immediately after incubation, samples were centrifuged at 1000 g for 10 min to obtain the supernatants. Aliquots were stored at − 70 °C until assay. Concentrations of IL-10, IL-6, IL-8, IL-1ra and TNF-α were determined in these culture supernatants. Monocyte counts were used to normalize cytokine production (difference between cytokine concentration in stimulated and unstimulated cultures) on a per cell basis [20].

Caffeine was measured in plasma by an HPLC method as previously described [16]. Adrenaline and cortisol were measured in plasma using commercially available enzyme-linked immunosorbent assay kits (Abnova Corporation, Taiwan) and (Elabscience Biotechnology Co, Ltd) respectively, with a spectrophotometric microplate reader (PowerWavei; BioTek, Winooski, VT). cAMP concentrations were determined in plasma using commercially available enzyme-linked immunosorbent assay kits (Arbor Assays) with a spectrophotometric microplate reader (PowerWavei; BioTek, Winooski, VT).

Cytokine concentrations were determined in plasma and in culture supernatants using commercially available enzyme-linked immunosorbent assay kits (Invitrogen, Carlsbad, CA, USA), with a spectrophotometric microplate reader.

### Statistical analysis

Statistical analysis was carried out using the Statistical Package for Social Sciences (IBM SPSS Statistics 23.0 for windows). The results are expressed as the means and standard deviations (S.D.), and *p* < 0.05 was considered statistically significant for all analysis. All the data were tested for their normal distribution (Shapiro-Wilk test). If the data were not normally distributed, statistical analysis was carried out on the logarithmic transformation of the data (cytokine, adrenaline, and cortisol concentrations). Exercise-related parameters (weight lost, maximum and average HR) under both conditions, placebo and supplemented, were compared using a t-test for unpaired data. Changes in blood variables during the study were analyzed using a time (pre-exercise, post-exercise, recovery) x condition (caffeine, placebo) within-between subjects ANOVA with repeated measures. Any significant F ratios subsequently shown were assessed using post hoc comparisons with Holm-Bonferroni correction for multiple comparisons applied to the unadjusted *p* value. Multiple linear regression models for IL-10 and IL-6 (logarithmic transformed) were also analyzed. Independent variables considered were time (pre-exercise, post-exercise, recovery), condition (caffeine, placebo) and adrenaline concentrations. The IL-10 model also included IL-6 as an independent variable.

## Results

### Characteristics of the main exercise trials

No differences were found between changes induced by exercise in the placebo and in supplemented conditions in weight loss (1.36 ± 0.43 vs. 1.38 ± 0.35 kg, *p* = 0.876), water intake during the test (271 ± 88 vs. 292 ± 102 mL, *p* = 0.627), average HR (159 ± 15 vs. 163 ± 15 beats·min^− 1^, *p* = 0.678) and maximum HR (168 ± 12 vs. 174 ± 12 beats·min^− 1^, *p* = 0.198).

### Plasma concentrations of caffeine, cAMP, adrenaline and cortisol

Plasma levels of caffeine and cAMP pre-exercise, post-exercise and after the 2-h recovery are shown in Table 2. Plasma caffeine was only detected in the supplemented condition after supplementation, with similar levels post-exercise and after recovery. cAMP plasma levels were only influenced by time (*p* < 0.001), with higher values post-exercise (*p* < 0.001).

**Table 2:** Changes in blood cell counts, plasma caffeine levels and cAMP during exercise and recovery following caffeine supplementation

	Pre-exercise	Post-exercise	Recovery	Main effect ***p***-values (T; C; TxC)
Caffeine (μg·mL^− 1^)				
Placebo	n.d.	n.d.	n.d.	< 0.001 < 0.001; < 0.001 ^a,b^
Caffeine	n.d.	8.99 ± 1.80 *	8.38 ± 2.40 *	
AMPc (μg·mL^−1^)				
Placebo	126.5 ± 15.5	174.5 ± 15.4	131.8 ± 19.2	< 0.001 ^a^; 0.327; 0.742
Caffeine	126.2 ± 17.4	178.8 ± 23.6	137.6 ± 21.4	
Leukocytes (10^3^·μL^− 1^)				
Placebo	6.21 ± 1.43	8.30 ± 1.68	8.80 ± 2.58	< 0.001; 0.002; 0.048 ^a,b^
Caffeine	6.66 ± 1.69	9.86 ± 2.51 *	10.2 ± 3.2 *	
Neutrophils (10^3^·μL^− 1^)				
Placebo	3.20 ± 0.68	4.51 ± 0.90	6.16 ± 1.83	< 0.001 ^a,b^; 0.002; 0.175
Caffeine	3.72 ± 1.08	5.64 ± 1.61	7.63 ± 2.38	
Lymphocytes (10^3^·μL^− 1^)				
Placebo	2.27 ± 0.53	3.00 ± 0.69	1.89 ± 0.40	< 0.001; 0.262; 0.006 ^a,b,c^
Caffeine	2.33 ± 0.49	3.34 ± 0.78 *	1.80 ± 0.36	
Monocytes (10^3^·μL^− 1^)				
Placebo	0.50 ± 0.11	0.54 ± 0.14	0.54 ± 0.14	0.028 ^a^; 0.383; 0.287
Caffeine	0.47 ± 0.09	0.59 ± 0.20	0.58 ± 0.21	

The values are the mean ± S.D. (*n* = 13)

*T* time (pre-exercise, post-exercise, recovery), *C* condition (placebo, caffeine), *TxC* interaction time x condition

*indicates significant differences between conditions at that time point

^a^indicates significant differences post-exercise vs. pre-exercise

^b^indicates significant differences recovery vs. pre-exercise

^c^indicates significant differences recovery vs. post-exercise

**Fig. 1:**
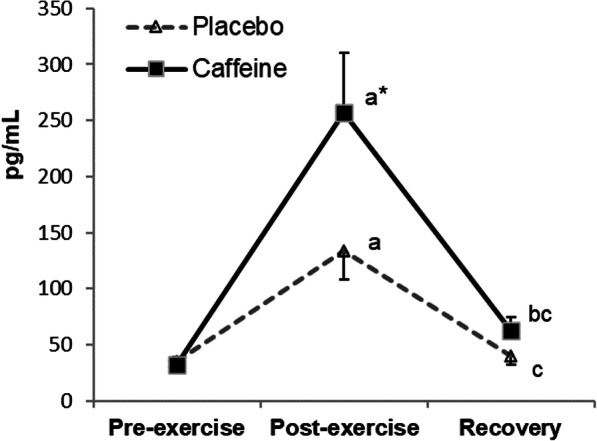
Changes in plasma adrenaline concentrations during exercise and recovery following caffeine supplementation. The values are the mean and S.D. (*n* = 13). *p* value (time): < 0.001; *p* value (condition): 0.030; *p* value (interaction): 0.029; * indicates significant differences between conditions at that time point; ^a^ indicates significant differences post-exercise vs. pre-exercise; ^b^ indicates significant differences recovery vs. pre-exercise; ^c^ indicates significant differences recovery vs. post-exercise

**Fig. 2:**
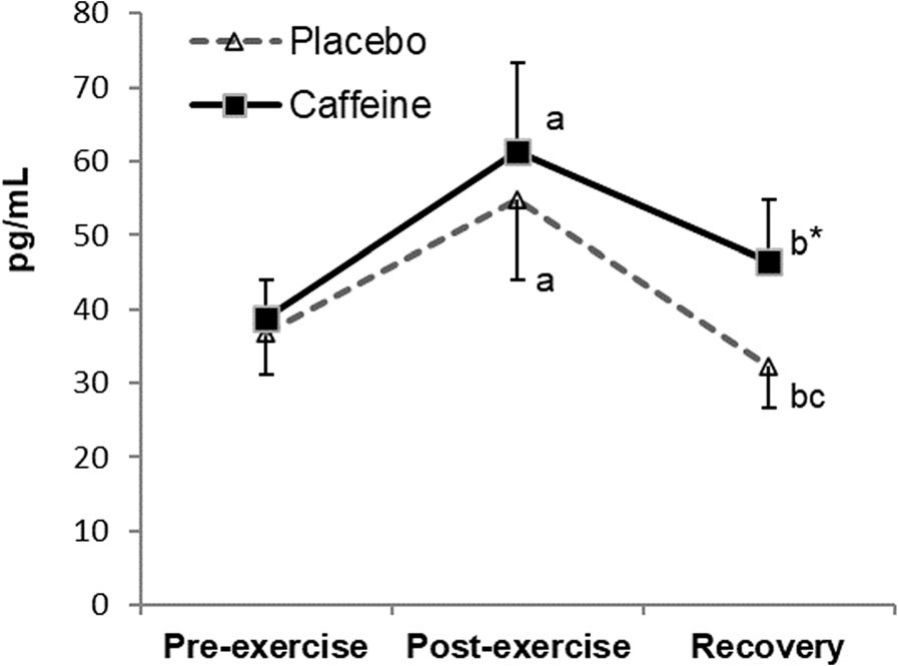
Changes in plasma cortisol concentrations during exercise and recovery following caffeine supplementation. The values are the mean and S.D. (*n* = 13). *p* value (time): < 0.001; *p* value (condition): 0.036; *p* value (interaction): 0.017; * indicates significant differences between conditions at that time point; ^a^ indicates significant differences post-exercise vs. pre-exercise; ^b^ indicates significant differences recovery vs. pre-exercise; ^c^ indicates significant differences recovery vs. post-exercise

A significant interaction (time x condition) for plasma adrenaline concentration (*p* = 0.029) was observed (Fig. 1). Plasma adrenaline increased post-exercise in both conditions (placebo *p* = 0.002; supplemented *p* = 0.003), with significantly higher values in the supplemented subjects (*p* = 0.030). A significant interaction was also found for plasma cortisol concentrations (*p* = 0.017) (Fig. 2). Post-recovery plasma cortisol concentrations were higher in the supplemented than in the placebo condition (*p* = 0.007), with values in the supplemented condition still higher than the pre-exercise values (*p* = 0.001).

### Circulating white blood cell counts

Table 2 also shows the changes in the leukocyte numbers after exercise and recovery. A significant interaction time x condition was found for the leukocyte number (*p* = 0.048). Leukocytes increased post-exercise (*p* < 0.001 for both conditions) and remained high during recovery. Values both post-exercise (*p* = 0.007) and post-recovery (*p* = 0.011) were significantly higher in the supplemented than in the placebo condition. A significant time x condition interaction was also found for the lymphocyte number (*p* = 0.006). The number of circulating lymphocytes increased significantly post-exercise (placebo *p* = 0.001, supplemented *p* < 0.001), with significantly higher values in the supplemented condition than in the placebo value (*p* = 0.021).

### Plasma cytokine concentrations

**Fig. 3:**
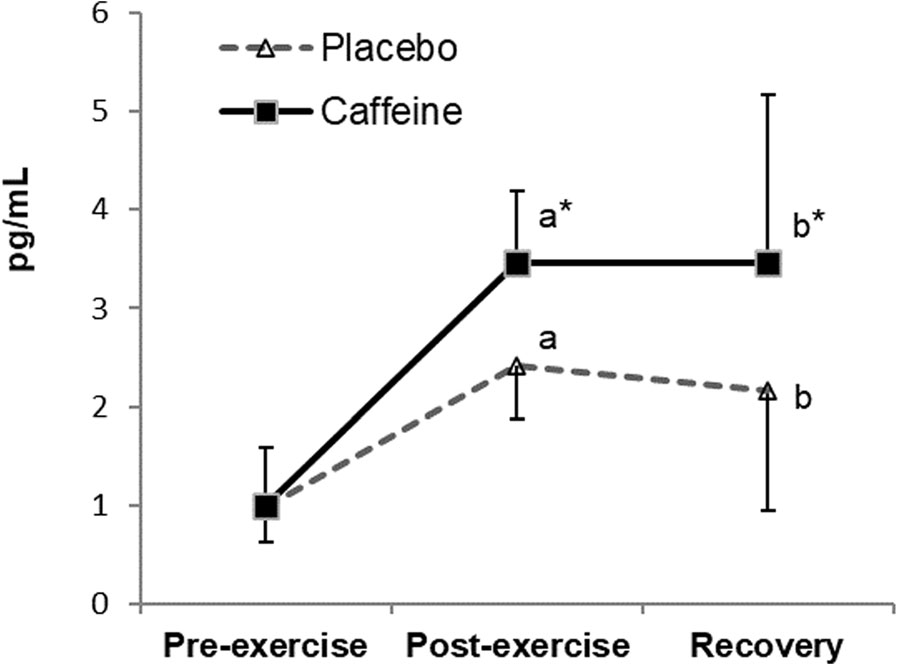
Changes in plasma IL-10 concentrations during exercise and recovery following caffeine supplementation. The values are the mean and S.D. (*n* = 13). *p* value (time): 0.003; *p* value (condition): 0.001; *p* value (interaction): 0.016; * indicates significant differences between conditions at that time point; ^a^ indicates significant differences post-exercise vs. pre-exercise; ^b^ indicates significant differences recovery vs. pre-exercise

A significant time x condition interaction (*p* = 0.016) was found for IL-10 plasma concentrations (Fig. 3). IL-10 levels increased post-exercise in both conditions (placebo *p* < 0.001, supplemented *p* = 0.001), with significantly higher values in the supplemented than in the placebo condition (*p* = 0.01). These values remained high after recovery (placebo *p* = 0.022, supplemented *p* = 0.006), with higher concentrations in the supplemented condition (*p* = 0.001).

**Fig. 4:**
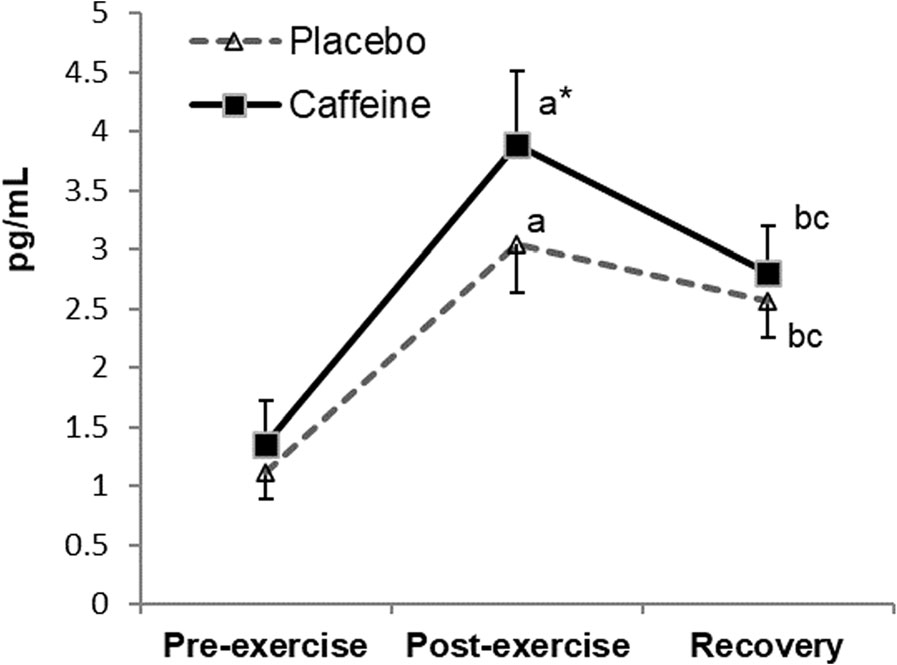
Changes in plasma IL-6 concentrations during exercise and recovery following caffeine supplementation. The values are the mean and S.D. (*n* = 13). *p* value (time): < 0.001; *p* value (condition): 0.011; *p* value (interaction): 0.017; * indicates significant differences between conditions at that time point; ^a^ indicates significant differences post-exercise vs. pre-exercise; ^b^ indicates significant differences recovery vs. pre-exercise; ^c^ indicates significant differences recovery vs. post-exercise

Figure 4 shows the changes in IL-6 levels in plasma. A significant time x condition interaction (*p* = 0.017) was found, with increased values post-exercise (placebo *p* = 0.002, supplemented *p* = 0.003) that were higher in the supplemented than those in the placebo condition (*p* = 0.003).

A significant effect of time was observed for IL-1ra, IL-4, IL-8, IL-12 and IFN-γ (Table 3), with increased post-exercise values for IL-1ra (*p* = 0.026), IL-4 (*p* = 0.001), IL-8 (*p* < 0.001), and IL-12 (*p* = 0.001).

**Table 3:** Changes in plasma cytokine concentrations during exercise and recovery following caffeine supplementation

	Pre-exercise	Post-exercise	Recovery	Main effect p-values (T; C; TxC)
IL-8 (μg·mL^− 1^)				
Placebo	48.6 ± 4.1	59.1 ± 5.0	50.2 ± 4.6	< 0.001 ^a^; 0.475; 0.290
Caffeine	49.8 ± 4.6	63.7 ± 4.4	48.6 ± 3.6	
IL-1β (μg·mL^− 1^)				
Placebo	17.1 ± 2.7	17.4 ± 2.7	14.6 ± 3.2	0.217; 0.600; 0.293
Caffeine	16.6 ± 2.7	19.6 ± 3.0	14.8 ± 1.8	
IL-12 (μg·mL^− 1^)				
Placebo	91.9 ± 6.6	113.1 ± 9.5	91.4 ± 15.1	0.007 ^a^; 0.563; 0.742
Caffeine	91.6 ± 7.8	117.7 ± 9.0	77.4 ± 4.4	
IL-1ra (μg·mL^− 1^)				
Placebo	2.21 ± 0.35	2.58 ± 0.52	2.30 ± 0.33	0.026 ^a^; 0.577; 0.307
Caffeine	2.28 ± 0.40	2.63 ± 0.34	2.18 ± 0.55	
IL-4 (μg·mL^− 1^)				
Placebo	1.74 ± 0.20	1.96 ± 0.21	1.56 ± 0.16	< 0.001 ^a^; 0.522; 0.070
Caffeine	1.60 ± 0.15	2.24 ± 0.24	1.60 ± 0.18	
IFN-γ (μg·mL^− 1^)				
Placebo	5.28 ± 0.41	6.10 ± 0.51	5.07 ± 0.67	0.015 ^a^; 0.493; 0.587
Caffeine	4.73 ± 0.44	6.03 ± 0.23	4.86 ± 0.27	

The values are the mean ± S.D. (*n* = 13)

*T* time (pre-exercise, post-exercise, recovery), *C* condition (placebo, caffeine), *TxC* interaction time x condition. Main effect for time

^a^indicates significant differences post-exercise vs. pre-exercise

### Multivariable linear regression analysis

Table 4 shows the three regression models obtained for IL-10. The regression analysis showed that IL-6 was the main predictor for IL-10 plasma concentrations (*p* < 0.001). Time (*p* = 0.001) and condition (*p* = 0.017) factors were also found to be significant predictors. No significant effect of adrenaline was found.

**Table 4:** Multivariable linear regression models for IL-10 with time, condition, adrenaline and IL-6 as independent factors

	Model 1		Model 2		Model 3	
	Coefficient	*p* value	Coefficient	*p* value	Coefficient	*p* value
**Input variable**						
Time			0.314	0.002	0.329	0.001
Condition					0.218	0.017
Adrenaline						n.s.
IL-6	0.547	< 0.001	0.416	< 0.001	0.379	< 0.001
**R**^**2**^	0.300		0.381		0.427	
**Adjusted R**^**2**^	0.290		0.364		0.404	
**F value**	32.496	< 0.001	23.070	< 0.001	18.396	< 0.001
**R**^**2**^ **change**	0.300		0.081		0.046	
**F change**	32.496	< 0.001	9.856	0.002	5.984	0.017

β values for significant predictors in each model are provided in the coefficient column

*n.s.* non-significant effect of this factor in any model

Logarithmic transformations of IL-10, IL-6 and adrenaline were used.

Table 5 shows the two regression models obtained for IL-6. Adrenaline was found to be the main predictor for IL-6 concentrations (*p* < 0.001). The factor time was also found to be a significant predictor (*p* < 0.001). No significant effect of the condition was found.

**Table 5:** Multivariable linear regression models for IL-6 with time, condition and adrenaline as independent factors

	Model 1		Model 2	
	Coefficient	*p* value	Coefficient	*p* value
**Input variable**				
Time			0.385	< 0.001
Condition				n.s.
Adrenaline	0.560	< 0.001	0.537	< 0.001
**R**^**2**^	0.314		0.462	
**Adjusted R**^**2**^	0.305		0.447	
**F value**	34.775	< 0.001	32.147	< 0.001
**R**^**2**^ **change**	0.314		0.148	
**F change**	34.775	< 0.001	20.567	< 0.001

β values for significant predictors in each model are provided in the coefficient column

*n.s.* non-significant effect of this factor in any model

Logarithmic transformation of IL-6 and adrenaline were used

### Stimulated cytokine production

When the stimulated cytokine production normalized to monocyte number values was considered (Table 6), an effect of time on the IL-1ra concentrations was observed (*p* = 0.002). The IL-1ra values post-recovery were higher than the pre-exercise (*p* = 0.005) and the post-exercise (*p* = 0.02) values.

**Table 6:** Changes in stimulated cytokine production during exercise and recovery following caffeine supplementation

	Pre-exercise	Post-exercise	Recovery	Main effect ***p***-values (T; C; TxC)
IL-10 (pg·10^3^ monocytes^− 1^)				
Placebo	1.11 ± 0.59	1.28 ± 0.66	1.36 ± 0.88	0.493; 0.644; 0.067
Caffeine	1.30 ± 0.79	1.43 ± 0.87	1.16 ± 0.67	
IL-6 (pg·10^3^ monocytes^− 1^)				
Placebo	242 ± 164	304 ± 224	310 ± 275	0.148; 0.744; 0.991
Caffeine	251 ± 152	312 ± 227	323 ± 251	
TNF-α (pg·10^3^ monocytes^− 1^)				
Placebo	13.1 ± 6.4	13.3 ± 6.2	13.7 ± 10.8	0.493; 0.255; 0.217
Caffeine	14.5 ± 7.0	10.8 ± 5.8	11.1 ± 6.1	
IL-1ra (pg·10^6^ monocytes^− 1^)				
Placebo	16.9 ± 12.8	21.7 ± 12.2	41.7 ± 32.4	0.002 ^a,b^; 0.458; 0.794
Caffeine	19.7 ± 9.4	24.2 ± 10.8	42.6 ± 28.4	
IL-8 (pg·10^3^ monocytes^− 1^)				
Placebo	17.6 ± 6.5	22.4 ± 11.6	20.1 ± 12.3	0.343; 0.312; 0.522
Caffeine	19.3 ± 7.3	20.3 ± 10.3	14.5 ± 8.9	

The values are the mean ± S.D. (*n* = 13)

*T* time (pre-exercise, post-exercise, recovery), *C* condition (placebo, caffeine), *TxC* interaction time x condition. Main effect for time

^a^indicates significant differences recovery vs. pre-exercise

^b^indicates significant differences recovery vs. post-exercise

## Discussion

The main finding of the present study was that caffeine supplementation induced higher IL-6 and IL-10 plasma concentrations in response to a treadmill exercise test performed at 70% VO_2_max. However, no effect of caffeine was observed in the responses of other anti-inflammatory (IL-1ra, IL-4) and pro-inflammatory (IL-12, IL-1β, IL-8, IFN-γ) cytokines.

In agreement with previous studies, IL-6 increased post-exercise [5, 9]. However, the increase observed was lower than those reported after longer and/or more intense exercises that compromise muscle glycogen availability [9]. In this study, a moderate exercise protocol performed by well-trained recreational athletes was used to highlight the effects of caffeine, avoiding the effects of muscle low energetic availability. Furthermore, this moderate exercise, rather than the strenuous ones, could reflect more properly a healthy exercise model. In agreement with a few previous studies [16, 17, 21, 22], higher IL-6 increases post-exercise in caffeine-supplemented athletes were observed in the present study. These higher increases in IL-6 after caffeine supplementation have been mainly attributed to a decreased clearance of IL-6 by the liver [15] due to the adrenaline-induced decrease in splanchnic blood flow [23]. In agreement with this suggestion, higher adrenaline concentrations were observed post-exercise in the supplemented participants, which could have been induced by this decreased clearance. Furthermore, the regression analysis showed that adrenaline was the main predictor for IL-6. These observations could confirm the adrenaline-induced decreased clearance as the main reason for increased IL-6 levels after caffeine supplementation.

As for IL-6, increases in IL-10 after exercise have been reported [24, 25], which is in agreement with the results of the present study. It is noteworthy that the increase in the caffeine-supplemented participants was higher than the increase observed in the placebo condition, which is in agreement with our previous results [16, 17]. A possible role for cAMP in these increased IL-10 levels was not found because no differences were observed between groups in post-exercise plasma cAMP levels. It has been reported that acute increases in IL-6 stimulate the production of IL-10 [10]. However, this mechanism has only been shown when IL-6 levels are much higher than the ones observed in this study [10]. Despite this last observation, in the present study, the regression analysis showed that IL-6 was the main predictor for IL-10 levels. Therefore, it seems that changes in IL-10 levels, including differences between conditions, could be attributed, at least in part, to IL-6 changes. It is noteworthy that the lack of differences between conditions in the IL-1ra response to exercise are not in agreement with this mechanism because it was suggested that IL-6 levels induce not only IL-10 but also IL-1ra production in response to exercise stimulus [10]. More studies are required to properly clarify this issue.

The possibility that higher IL-10 levels post-exercise in caffeine-supplemented participants were due to a decrease in IL-10 clearance was also tested using regression analysis. In this regard, it has been reported that the kidney contributes significantly to IL-10 clearance [26, 27], and adrenaline decreases renal blood flow [28]. Therefore, the higher adrenaline levels in the caffeine-supplemented participants could lead to a lower IL-10 renal clearance and, therefore, to higher plasma levels in this supplemented condition. However, the regression analysis performed showed that IL-10 levels were not related to adrenaline. Therefore, it seems that, in contrast with IL-6, this mechanism would not contribute significantly to increased IL-10 levels in the caffeine condition.

Recent reports have suggested that caffeine could exert an anti-inflammatory effect by inhibiting acetylcholinesterase activity and therefore, maintaining higher levels of acetylcholine, a suppressor of pro-inflammatory cytokines [29–31]. The results obtained in the present study did not support this mechanism because the response of pro-inflammatory cytokines, such as IL-8 and IFN-γ, to exercise was similar in the supplemented and in the placebo conditions. Thus, it seems that at least in response to this moderate exercise, the mechanisms leading to the suggested caffeine anti-inflammatory effects would be related to higher IL-10 levels rather than to an inhibition of pro-inflammatory cytokines. It is worth mentioning that higher IL-10 levels in caffeine-supplemented participants could suppose a healthier anti-inflammatory effect of exercise. In this regard, it has been suggested that the healthy anti-inflammatory effects of physical activity counteracting low-grade systemic inflammation are due in part to the increased release of anti-inflammatory cytokines, such as IL-10, after each acute exercise session [32]. Higher cortisol levels in the caffeine-supplemented participants found in the present study could also suppose a higher anti-inflammatory effect because it has been reported that cortisol exerts potent anti-inflammatory actions [33].

In the present study, no effects of caffeine supplementation on the LPS-stimulated in vitro cytokine production post-exercise were observed. It is noteworthy that only a few previous studies have used similar approaches when caffeine effects during exercise were analyzed [22, 34, 35], but none of them have yet measured cytokine-stimulated production. The results obtained in the present study could be in agreement with the lack of effect of caffeine supplementation on the blood mononuclear cell cytokine levels in response to exercise observed previously [17]. Several in vitro studies have shown that caffeine can modulate various aspects of both innate and adaptive immunity, including cytokine production by different cellular lines and tissues [13]. However, these studies used caffeine concentrations much higher than the physiological ones and the ones present in the blood cell cultures in the present study (approximately 3 μg/mL or 15 μM). The very slight effect of exercise on stimulated cytokine production is in agreement with previous reports [20] and, among other factors, could reflect the not-very demanding characteristic of the exercise performed.

Some limitations of this study should be acknowledged. This study considered only males; it would be interesting to analyze the effects of caffeine supplementation in females and in older participants. Furthermore, it would also be interesting to determine whether the effects of caffeine observed in the present study were maintained in exercise tests of different durations and/or intensities, mainly in more demanding exercises. More studies are needed to determine the sources of increased cytokine concentrations post-exercise, mainly the source of higher IL-10 levels in caffeine-supplemented participants.

## Conclusions

Caffeine supplementation induced higher post-exercise circulatory levels of IL-10 and IL-6. Those caffeine-induced higher IL-10 levels could suppose an enhancement of the anti-inflammatory and health-enhancing attributes of exercise; nonetheless, more studies are necessary to determine the concrete mechanisms leading to these increased levels and their consequences. The lack of caffeine effects on other cytokines and/or stimulated cytokine production did not allow the establishment of a general anti-inflammatory effect of caffeine.

## Data Availability

The datasets used and/or analyzed during the current study are available from the corresponding author on reasonable request.

## Abbreviations

cAMP: Cyclic adenosine monophosphate
IL: Interleukin
LPS: Lipopolysaccharide
VO_2_max: Maximal oxygen uptake
HR: Heart Rate
MET: Metabolic equivalents
BMI: Body Mass Index
EDTA: Ethylenediaminetetraacetic acid
ANOVA: Analysis of variance
